# Supplementary material for: Utilization of borax and its impact on the income and the livelihood of miners and other stakeholders: A case of Uganda
Source: PLoS One. 2025 Aug 19;20(8):e0320543. doi: 10.1371/journal.pone.0320543 (PMC12364340; doi:10.1371/journal.pone.0320543)
Supplement: Attachment — Submitted filename: Response to Reviewers.pdf [file pone.0320543.s003.pdf]

MAKERERE UNIVERSITY
P.O. Box 7072 Kampala Uganda
E-mail: dceh@musph.ac.ug
Tel: 256-41-4-543872/031-2-263158
Fax: 256-41-531807
Website: http://www.musph.ac.ug

SCHOOL OF PUBLIC HEALTH
Department of Disease Control & Environmental Health

04/04/2025

Editor

PLOS ONE

RE: SUBMISSION OF THE REVISED MANUSCRIPT TO YOUR JOURNAL

Thank you so much for your speedy consideration of our manuscript. We have to the best of our ability tried to rectify according to your valued comments from the reviewers.

I am enclosing herewith a revised manuscript entitled "**Utilization of borax and its impact on the income and the livelihood of miners and other stakeholders : A case of Uganda.**"

Please, find below our comments summarized in a table.

I look forward to your positive response.

Sincerely,

Mr. James Natweta Baguma

Research Associate
Department Disease Control and Environmental Health.
Makerere University School of Public Health
Tel: +256775989895 Email: bagumajamesnat@gmail.com

**REMARKS**	**STATUS**	**LOCATION**
1. Please ensure that your manuscript meets PLOS ONE's style requirements, including those for file naming	We have revised it to ensure that it meets the requirements	The whole manuscript
2. We note that the grant information you provided in the 'Funding Information' and 'Financial Disclosure' sections do not match.<br>When you resubmit, please ensure that you provide the correct grant numbers for the awards you received for your study in the 'Funding Information' section. Thank you for stating the following in the Acknowledgments Section of your manuscript:<br>"The Uganda National Association of Community and Occupational Health (UNACOH), with funding support from Dialogos, a Danish Non-Government Organization supported by Danish Government conducted a study in Buhweju, Kassanda and Busia districts under the Free Your Mine project (FYM 2025) to assess effects of mercury-free gold processing on the incomes and livelihoods of artisanal and small-scale gold miners and stakeholders in Buhweju, Busia and Kassanda districts in Uganda."<br>We note that you have provided funding information that is not currently declared in your Funding Statement. However, funding	We have edited the Funding Information Section in the system	Funding Information Section in the system

information should not appear in the Acknowledgments section or other areas of your manuscript. We will only publish funding information present in the Funding Statement section of the online submission form.<br>Please remove any funding-related text from the manuscript and let us know how you would like to update your Funding Statement. Currently, your Funding Statement reads as follows:<br>"The author(s) received no specific funding for this work"<br><br>Please include your amended statements within your cover letter; we will change the online submission form on your behalf.		
4. When completing the data availability statement of the submission form, you indicated that you will make your data available on acceptance. We strongly recommend all authors decide on a data sharing plan before acceptance, as the process can be lengthy and hold up publication timelines. Please note that, though access restrictions are acceptable now, your entire data will need to be made freely accessible if your manuscript is accepted for publication. This policy applies	We have also assured the journal that the data will be available and accessible.	Line 607

to all data except where public deposition would breach compliance with the protocol approved by your research ethics board. If you are unable to adhere to our open data policy, please kindly revise your statement to explain your reasoning and we will seek the editor's input on an exemption. Please be assured that, once you have provided your new statement, the assessment of your exemption will not hold up the peer review process.		
5. Please ensure that you refer to Figure 1-3 in your text as, if accepted, production will need this reference to link the reader to the figure.	We have revised the manuscript to include clear references to Figures 1–3 within the main text including Figure 4	Line 155, 199, 239, and 440
6. We note that Figure 1-3 includes an image of a participant. As per the PLOS ONE policy (http://journals.plos.org/plosone/s/submission-guidelines#loc-human-subjects-research) on papers that include identifying, or potentially identifying, information, the individual(s) or parent(s)/guardian(s) must be informed of the terms of the PLOS open-access (CC-BY) license and provide specific permission for publication of these details under the terms of this license. Please download the Consent Form for Publication in a PLOS Journal (http://journals.plos.org/plosone/s/file?id=8ce	The individuals in this manuscript have given written informed consent to the study team. This has been also made clear in the methods section under the ethics statement.	In the signed consent form

6/plos-consent-form-english.pdf). The signed consent form should not be submitted with the manuscript but should be securely filed in the individual's case notes. Please amend the methods section and ethics statement of the manuscript to explicitly state that the patient/participant has provided consent for publication: "The individual in this manuscript has given written informed consent (as outlined in PLOS consent form) to publish these case details".<br><br>If you are unable to obtain consent from the subject of the photograph, you will need to remove the figure and any other textual identifying information or case descriptions for this individual.		
Reviewer #1: Article is well structured, well written and technically sound. Authors should simply check again for any minor grammatical errors and correct them. e.g., line 337 and 338 (change 'its' to it; 'so' into to).	We have conducted a thorough grammar and typographic review. Specific corrections have been made.	Line 353, 354
Reviewer #2: The manuscript comes out as an advocacy for the use Borax in gold processing, without much justification. Borax itself has not been described/ defined and there does not appear to be any significant	We revised the manuscript to remove any advocacy tone and ensure objectivity. The discussion now	Introduction section

technical references on the efficacy of Borax. There is clearly limited uptake of Borax with artisanal miners in the study preferring the use of mercury in gold processing, instead. One quoted statistic says that in a field demonstration, Borax recovered 40% more gold than mercury, but the experiment itself is not properly described. More examples of properly set-up experimentations, or of actual mine recovery statistics may help lend more objectivity to the study. It appears the stakeholders are saying that mercury is a faster and cheaper way of gold recovery than mercury. However, even in the discussion, the authors hold on to a 'truth' that Borax use is 'less time-consuming'.<br><br>The tone of the manuscript throughout should refrain from obvious biases and be more objective; the authors should let the data speak for themselves.<br><br>Although generally well-written, the manuscript has some typographic errors (e.g. P in sample size calculation is applied as 12%, but stated as 50% in the list of variables) and repetitiveness (e.g. in the Discussion) which need to be rectified throughout.	emphasizes findings rather than inferred benefits.<br><br>We have added a clear definition and background on borax in the Introduction and expanded references on its efficacy based on global studies.<br><br>Details of the field demonstration, including methodology and participant numbers, have been elaborated in the Methods section.<br><br>The discrepancy has been corrected; "P" is now consistently and correctly defined across the manuscript.	Line 190<br><br>Line 181
This study represents a substantial mixed-methods effort addressing a globally significant issue: the use of mercury by artisanal gold	We have revised the results section to enhance clarity	In the results section and

miners—a metal highly toxic to both human health and the environment.<br>However, the manuscript requires a thorough revision of the presentation of results and a structural reworking of the article to make it clearer and more accessible to readers.<br>The stated objective of the article is to “assess the impact of borax utilization in gold processing on the income and livelihoods of miners and stakeholders in Buhweju, Busia and Kassanda districts in Uganda where the Free Your Mine project is being implemented.” However, the content of the article suggests that it primarily evaluates the barriers and facilitators to borax use within this population. Therefore, we recommend that the objective be reformulated to more accurately reflect the actual focus of the study.<br>Additionally, terminology should be used consistently throughout the manuscript:<br>• borax should be written in lowercase;<br>• Abbreviations should not be defined multiple times.	and coherence. The presentation now follows a more logical structure, beginning with descriptive data and then analyzing key themes around barriers, facilitators, and perceived outcomes of borax use.<br><br>We have thoroughly reviewed the manuscript to ensure consistent usage of terminology throughout. *borax* is now written in lowercase uniformly across the text unless where it is starting the sentence.<br><br>All abbreviations are now defined only once, upon first mention, and have been removed where redundantly redefined.<br>for your valuable suggestions.	entire manuscript
Introduction:<br>• Clearly present, based on the literature, the benefits and drawbacks of using borax.<br>• The "Free Your Mine" project needs to be better explained: objectives, context, and	We have expanded the description of the “Free Your Mine” (FYM) project within the Introduction.	In the Introduction section

implementation sites.<br>• The data from Ghana, mentioned later in the article, would be more appropriately integrated into the discussion section, where they can help contextualize the findings.	We have revised the Introduction to clearly present both the benefits and limitations of using borax in artisanal gold processing, based on relevant literature	
Methods:<br>• Add a map to indicate the inclusion areas.<br>• Specify the number of participants per site.<br>• Indicate how many participants were trained in mercury-free techniques.<br>• Review the mathematical formulas:<br>o The "P" used does not refer to the prevalence as claimed.<br>o It would be preferable to clearly explain the calculation in plain language.<br>• Provide more context on ASGM (artisanal and small-scale gold mining) in Uganda: are these activities legal or not?<br>• Lines 173–176: it is unclear whether the KIIs involved 12 groups or 5.<br>• Define what is meant by "youth mining groups."<br>• The sampling method needs to be explained more clearly.<br>• Line 205: specify whether the qualitative questionnaires were structured or semi-structured, and include the questionnaire in an	• A map showing study areas has been added.<br>• Participant numbers per site and training status are now specified.<br>• KIIs and youth group definitions are clarified.<br>• The qualitative tools have been attached in the appendix.<br>• Mathematical formulas and sampling method explanations are now simplified for clarity.	Line 166<br><br>Line 190<br><br>Line 193 to 199<br><br>Another Attachment<br><br>Line 174-188

appendix.		
Results:<br>• Lines 266–269 should be removed.<br>• The denominators used for ratio calculations must be clearly presented. Since some questions were conditional, readers need to follow how the calculations were made.<br>• Table 4 should show the sample size in each category.<br>• All table titles should be placed above the tables.<br>• Clearly separate descriptive data from the quantitative and qualitative components. For instance, the profile of focus group participants is unclear.<br>• Line 296: 21.7% does not constitute a "notable majority."<br>• Line 299: 1.9% of participants use borax for gold extraction — which equates to only 3 individuals? Clarify the distinction between purification and extraction, since the key issue is replacing mercury in the extraction process.<br>o How were participants categorized as borax users or not with such a low number? This represents a major bias in the study.<br>o Hence the need to reformulate the study objective accordingly.<br>• A flow chart should be added, showing:<br>o Number of participants trained vs. untrained;<br>o Their behavior in terms of borax use vs.	• Descriptive and analytical data have been clearly separated.<br>• Tables have been revised with sample sizes, corrected titles/locations, and denominators.<br>• Barriers to borax adoption are now explicitly reported in the Results section.<br>• Currency values are presented in both UGX and USD.<br>• All inconsistencies in interpretation (e.g., Line 322, Line 364) have been addressed.<br>• The titles have all been adjusted to match the content<br>• The phase accepted has been adjusted to affirmed<br>• The other method is	In the Results section<br><br>All tables have been revised<br><br>Barriers are mentioned in the qualitative results<br><br>All currency values<br><br>In the results section

mercury or other methods.<br>• Assess whether borax use varies depending on the location (e.g., proximity to the Free Your Mine project site).<br>• Data on borax or mercury use should be accompanied by a time frame/unit (cf. Line 301, Table 3, Line 390, Table 8...).<br>• Present monetary amounts in Ugandan shillings with USD equivalents.<br>• Line 322: the interpretation provided does not match the content of the referenced table, which is titled "Likelihood of continuing to use mercury after learning about gravity/borax method."<br>• Barriers to borax use are not presented in the results section, despite being a central takeaway of the study. They are only discussed later, which is a limitation.<br>• Line 364: the table does not match the title.<br>• Line 366: check for possible errors in the standard deviation (SD).<br>• Line 388: the phrase "accepted that" is unclear — please rephrase.<br>• Line 392: clarify what other methods (besides mercury or borax) are being referred to.<br>• Table 8: if a respondent used mercury more than 12 years ago, should they still be counted as a current user?	using water only without other chemicals.<br><br>• The unit of measurement of time was in months since the mercury technology is also a new technology in Uganda thus only respondents who had used Mercury for the last 12 months were considered.	
**Discussion:**<br>• Start with a brief summary of the main findings, before discussing them.	• Begins with a summary of key findings.	Line 451 to 456

• Clearly present the limitations of the study.<br>• Discuss the results in relation to the regional context and existing literature.<br>• The literature-based benefit-risk analysis of borax should be placed in the introduction, not in the discussion.<br>• Compare your findings with those from other countries, such as Ghana.<br>• Line 435: not clearly worded — rephrase.<br>• Lines 441–442: these elements should appear in the introduction.<br>• Lines 462–464: same comment.<br>• Lines 475–495: limited relevance — this section could be condensed to two sentences.<br>• Lines 558–562: it is uncertain whether these conclusions are fully supported by the results.<br>• Line 572: clarify what is meant by "acetylen gas", or remove the reference.<br>Finally, the article should clearly discuss the barriers to borax adoption, as well as measures to engage communities in transitioning to mercury-free gold mining.<br>Is the main issue access to borax, water availability, lack of training, or time required for processing? These points should be clarified.	• Now integrates comparison with other studies.<br>• Risk-benefit analysis of borax is moved to the Introduction.<br>• Limitations are acknowledged explicitly.<br>• Unclear or less relevant content has been condensed or rephrased (e.g., Lines 435, 441–442, 475–495, 558–562).<br><br>• "Acetylen gas" is well known	Throughout the Discussion<br><br>In the Introduction section<br><br>Line 484 to Line 500<br>In regard to gold extraction, Acetylen gas is used in conjunction with borax and is employed to quickly melt and coalesce the gold concentrate
