# Supplementary material for: Utilization of borax and its impact on the income and the livelihood of miners and other stakeholders: A case of Uganda
Source: PLoS One. 2025 Aug 19;20(8):e0320543. doi: 10.1371/journal.pone.0320543 (PMC12364340; doi:10.1371/journal.pone.0320543)
Supplement: Attachment — Submitted filename: Response to Reviewers-.pdf [file pone.0320543.s005.pdf]

M A K E R E R E U N I V E R S I T Y
P.O. Box 7072 Kampala Uganda
E-mail: dceh@musph.ac.ug
Tel: 256-41-4-543872/031-2-263158
Fax: 256-41-531807
Website: http://www.musph.ac.ug

SCHOOL OF PUBLIC HEALTH
Department of Disease Control & Environmental Health

12/06/2025

Editor

PLOS ONE

RE: <u>SUBMISSION OF THE REVISED MANUSCRIPT TO YOUR JOURNAL</u>

Thank you so much for your speedy consideration of our revised manuscript. We have to the best of our ability tried to rectify according to your valued comments from the reviewers.

I am enclosing herewith a revised manuscript entitled "**Utilization of borax and its impact on the income and the livelihood of miners and other stakeholders: A case of Uganda.**"

This study was funded by Dialogos, a non-governmental organization (NGO) based in Denmark. through the Uganda National Association of Community and Occupational Health. (UNACOH). The support from Dialogos was instrumental in enabling the successful implementation of the project activities.

Please, find below response summarized in a table.

I look forward to your positive response.

Sincerely,

Mr. James Natweta Baguma

Research Associate
Department Disease Control and Environmental Health.
Makerere University School of Public Health
Tel: +256775989895 Email: bagumajamesnat@gmail.com

REMARKS	STATUS	LOCATION
1. Defined borax and explained its use and adequately corroborated its efficacy	We have defined and explained borax and its use as well as its efficacy	Line 76-79<br><br>Line 81-89
2. Provided details of a specific experiment which supposedly showed that borax is 40% more efficient than mercury in gold recovery.	The details of this experiment are well highlighted in the study conducted by Stoffersen B et al titled Comparison of gold yield with traditional amalgamation and direct smelting in artisanal small-scale gold mining in Uganda. Journal of Health and Pollution. 2019 Dec 1;9(24):191205.	Reference 8
3. Adequately replaced advocacy with objectivity throughout the article.	We have adjusted the language	Throughout the manuscript
